# Review of Adult Critical Care Calls to the Out-of-Hours Resident Pharmacist Service: A Retrospective Single-Center Observation Study

**DOI:** 10.7759/cureus.83112

**Published:** 2025-04-28

**Authors:** Jiaxian Wang, Janique Waghorn, Stephanie Khoo, Hementiben Patel, Christopher Remmington

**Affiliations:** 1 Institute of Pharmaceutical Science, School of Cancer and Pharmaceutical Sciences, King's College London, London, GBR; 2 Pharmacy and Adult Critical Care, Guy's and St Thomas' NHS Foundation Trust, London, GBR

**Keywords:** critical care units, hospital pharmacy services, intensive care, on-call, on-call pharmacist, out-of-hours, resident pharmacist

## Abstract

Background

There is good evidence describing on-call pharmacy services internationally; however, there is limited evidence evaluating out-of-hours (OOH) calls from adult critical care units. Our primary objective is to describe the characteristics of OOH calls from adult critical care units to resident pharmacists, to inform service provision to this high-risk specialty. Our secondary objective is to compare the characteristics of calls during evening and nighttime hours.

Methods

This is a retrospective observational review of adult critical care OOH calls received by resident pharmacists from August 2023 to July 2024 at a large tertiary center. OOH calls were defined as calls received from 5 pm to 9 am Monday to Sunday, and we further differentiated the calls into evening calls (5 pm to 9 pm) and nighttime calls (9 pm to 9 am). We collected the characteristics of OOH calls, i.e., patient age and sex, ward location, type of query (medicines information/advice or supply), drug requested, category of drug, route of administration, day of the week, date and time of call received, and duration to resolution of call. We compared the characteristics of OOH calls during the evening (5 pm to 9 pm) vs. nighttime (9 pm to 9 am) using an independent samples t-test for continuous data and Pearson's χ^2^ test for categorical data.

Results

We identified 5085 OOH calls from adult critical care units, which represented 15.3% of all calls received by the resident pharmacist during the study period. Of these, 2080 calls were received between the hours of 5 pm and 9 pm, and 3004 calls were received between the hours of 9 pm and 9 am. Most calls related to male patients (n=2364; 46.5%), with a mean (SD) of 56.6 (17.5) years. The highest number of calls was received from ICU1 (n=1183; 23.3%), followed by ICU2 (n=923; 18.2%) and ICU6 (n=787; 15.5%). The greatest proportion of calls was received on a Thursday (n=806; 15.9%), and January 2024 was the busiest month for calls (n=528; 17.6%). Most calls were for medication supply (n=4885; 96.1%), and most calls took 50 minutes and over to complete (n=4724; 92.9%). We compared the general characteristics of calls between 5 pm and 9 pm with calls between 9 pm and 9 am and found no significant difference in the proportion of calls to individual critical care units (p=0.050), the day of the week (p=0.513), or the category of the call (p=0.074). We found no significant difference in patient characteristics when comparing OOH calls received during the evening and nighttime periods: male sex 48.4% vs. 45.2% (p=0.563) and age 56.3 (17.6) vs. 56.9 (17.5) years (p=0.250). There were 5915 medication requests for supply or advice during the study period. Total parenteral nutrition was the most requested item (n=255; 4.3%), and the most requested category of medicine was anti-infectives (n=921; 18.1%). We compared the characteristics of medicines between evening and nighttime and found a higher proportion of calls during nighttime for all classes of drugs (p<0.001) and most routes of administration (p<0.001).

Conclusions

OOH pharmacy services to adult critical care provide timely supply and advice of critical medicines; however, no conclusions of impact on patient outcomes can be inferred. Further work should explore the impact of calls on avoidable patient harm, the economic analysis of the resident pharmacist on-call service, and whether the integration of artificial intelligence decision support could lower call volume.

## Introduction

Pharmacy departments provide out-of-hours (OOH) services to ensure timely access to critical medicines and advice internationally [1-3]. However, variations in OOH pharmacy service provision exist, particularly in the United Kingdom, with few centers providing an on-site resident pharmacist [2,4]. Limited OOH pharmacy services may contribute to omitted doses of medicines, delayed discharges, and inadequate transfer of care [5].

Guy's and St Thomas' NHS Foundation Trust is a large tertiary center in the United Kingdom, providing care for a population of 2.8 million patients, with one of the largest critical care services in the United Kingdom and one of London's busiest emergency departments [6]. The core working hours for adult critical care pharmacists are 8:30 am to 5:00 pm Monday to Friday [2]. At weekends, adult critical care pharmacists provide a reduced on-site service from 9 am to 5 pm on Saturday and Sunday. OOH is typically defined as the delivery of pharmacy services outside of these standard working hours [2]. The Pharmacy Department at Guy's and St Thomas' NHS Foundation Trust also provides a resident pharmacist service 24 hours a day, seven days per week, as an emergency service, comprising foundation-level pharmacists.

The OOH resident pharmacist service aims to support healthcare professionals to optimize patient outcomes by advising on the effective use of medicines and avoiding omissions or delays in treatment through the supply of critical medicines as outlined in the Professional Standards for Hospital Pharmacy Services [7]. The resident pharmacist service is particularly important for adult critical care areas given the acuity and complexity of this patient cohort [8]. Critical care patients are complex due to the pharmacokinetic-pharmacodynamic changes in critical illness, the need for dynamic dose optimizations in organ dysfunction and extracorporeal support (renal replacement therapy and extracorporeal membrane oxygenation), and the frequent route of administration changes to medicines [9]. The resident pharmacist service includes the emergency supply of non-stock medicines, supply of controlled drugs, and medication advice and optimization to mitigate adverse drug events [10,11]. Queries from adult critical care units are received through a web-based and smartphone system Smartpage® (Alcidion, South Yarra, Victoria, Australia) [12]. The Pharmacy Department also provides an off-site on-call support service to assist resident pharmacists with more complex medication queries, including dose optimizations in critical illness, approval to supply restricted medicines, and drug-drug compatibility issues [4]. The pharmacists providing this on-call support service range from foundation to advanced level [13,14]. 

Existing literature describes general pharmacy OOH services in acute hospitals; however, there is no data reporting the characteristics of OOH calls to resident pharmacists from adult critical care units [1,15,16]. Our primary objective was to describe the characteristics of OOH calls from healthcare professionals on adult critical care units to the on-call resident pharmacists, to inform service provision to this high-risk specialty. Our secondary objective was to compare characteristics of calls during evening and nighttime hours.

## Materials and methods

Study design and sample

A retrospective review was conducted on all OOH calls received by resident pharmacists from adult critical care from August 2023 to July 2024 at Guy's and St Thomas' NHS Foundation Trust, London, England. We extracted a log of OOH calls and messages received by the resident pharmacist from healthcare professionals working in adult critical care units using Smartpage® analytics dashboard during the study window and exported it into Microsoft Excel (Microsoft Corporation, Redmond, Washington, United States) for analysis [12]. Calls and messages received by non-resident pharmacists and from healthcare professionals on wards outside of adult critical care units were removed from the search strategy and excluded from the study. Smartpage® is an advanced smartphone and web-based system for hospital communication and task management used by resident pharmacists to log all calls and messages received from healthcare professionals relating to medicines during their working shift in our institution [12].

OOH calls were defined as calls received from 5 pm to 9 am Monday to Sunday, and we only included the calls during this time period. We further differentiated the calls into evening calls (5 pm to 9 pm) and nighttime calls (9 pm to 9 am) as these time periods represented separate resident pharmacist shift patterns. Each call was categorized as an evening or nighttime call on the Microsoft Excel database to allow for comparisons between the two time points. We collected patient age and sex, characteristics of calls including critical care unit location, type of medicine query (medicine information/advice or supply), medicine requested, category of medicine, route of administration of medicine, day of the week of the call, date and time of call received, and duration to resolution of call.

Critical care unit characteristics

Guy's and St Thomas' NHS Foundation Trust is a tertiary academic hospital with one of the largest adult critical care services in the United Kingdom. The annual patient volume to adult critical care units exceeds 5000 admissions per year. The adult critical care service delivers care to a plethora of specialties, including emergency medical admissions, hematology and oncology patients, and peri- and postoperative care for elective and emergency cardiothoracic, head and neck, renal and pancreatic transplant, vascular, upper gastrointestinal, and urological patients. Additionally, there is a dedicated severe respiratory failure unit, including a regional extracorporeal membrane oxygenation service, and a long-term respiratory failure unit. The study included patients admitted to the following critical care units: Intensive Care Unit (ICU) 1, ICU 2, ICU 6, Guy's Critical Care Unit (GCCU), Overnight Intensive Recovery (OIR), Victoria High Dependency Unit (VHDU), and Lane Fox Unit (LFU).

Statistical analysis

The development of figures and tables was completed in Microsoft Excel (Version 16.92), and data were anonymized during extraction from the Smartpage® analytics dashboard. Categorical data are represented as counts and proportions, while continuous data are presented as means and standard deviations (SD). We performed a complete analysis of all calls and messages, with no treatment of missing data. We compared the characteristics of OOH calls during the evening (5 pm to 9 pm) versus nighttime (9 pm to 9 am) using an independent samples t-test for continuous data and Pearson's χ^2^ test for categorical data. All tests were two-tailed, with p<0.05 considered statistically significant. Assumptions testing was met for the independent samples t-test and expected counts for Pearson's χ^2^ test. We performed no adjustment for potential confounding factors. Data was analyzed using RStudio (Version 2024.04.2+764, R Foundation for Statistical Computing, Vienna, Austria) and IBM SPSS Statistics for Windows, Version 29.0.2.0 (Released 2023; IBM Corp., Armonk, New York, United States).

Ethics approval

Institutional approval was gained from the Quality Improvement and Safety Committee at Guy's and St Thomas' NHS Foundation Trust (approval number: 16797; date of approval: November 28, 2024). The need for review by a research ethics committee and informed consent was waived as the study met the criteria of a service evaluation.

## Results

General characteristics

**Figure 1:**
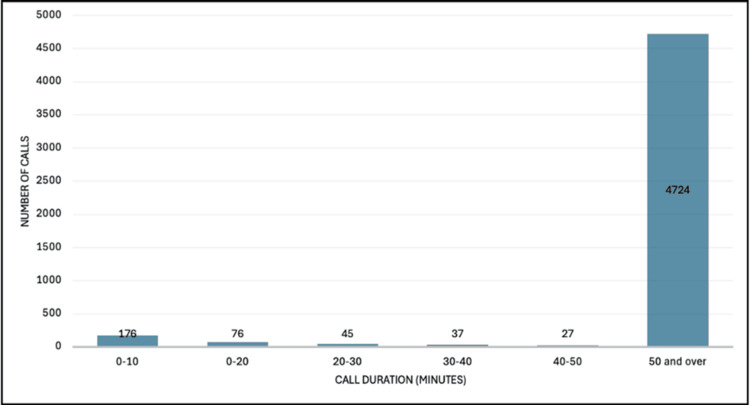
Analysis of completion times of adult critical care out-of-hours calls received by resident pharmacists

We identified 5085 OOH calls from adult critical care units, which represented 15.3% of all calls received by the resident pharmacist during the study period. Of these, 2080 calls were received between the hours of 5 pm and 9 pm, and 3004 calls were received between the hours of 9 pm and 9 am. Most calls related to male patients (n=2364; 46.5%), with a mean (SD) of 56.6 (17.5) years (Table 1). The highest number of calls was received from ICU1 (n=1183; 23.3%), followed by ICU2 (n=923; 18.2%) and ICU6 (n=787; 15.5%) (Table 1). The greatest proportion of calls was received on a Thursday (n=806; 15.9%), and January 2024 was the busiest month for calls (n=528; 17.6%) (Table 1). Most calls were for medication supply (n=4885; 96.1%), and most calls took 50 minutes and over to complete (n=4724; 92.9%) (Figure 1). We compared the general characteristics of calls between 5 pm and 9 pm with calls between 9 pm and 9 am and found no significant difference in the proportion of calls to individual critical care units (p=0.050), the day of the week (p=0.513), or the category of the call (p=0.074). We found no significant difference in patient characteristics when comparing OOH calls received during the evening and nighttime periods: male sex 48.4% vs. 45.2% (p=0.563) and age 56.3 (17.6) vs. 56.9 (17.5) years (p=0.250).

**Table 1:** Characteristics of critical care out-of-hours calls received by resident pharmacists Continuous data expressed as mean (standard deviation); categorical data as counts (% of counts). Continuous data compared using the independent samples t-test; categorical data using Pearson's χ^2^ test. GCCU: General Critical Care Unit; ICU1, ICU2, ICU6: Intensive Care Unit 1, 2, 6; LFU: Lane Fox Unit; OIR: Overnight Intensive Recovery; VHDU: Victoria High Dependency Unit

Characteristic	All	5 pm to 9 pm	9 pm to 9 am	P-value
Sex				
Male	2364 (46.5)	1007 (48.4)	1357 (45.2)	0.563
Female	1408 (27.7)	586 (28.2)	822 (27.4)	
Not known	1312 (25.8)	487 (23.4)	825 (27.5)	
Age (years)	56.6±17.5	56.3±17.6	56.9±17.5	0.250
Proportion of calls by critical care ward				
ICU1	1183 (23.3)	507 (24.4)	676 (22.5)	0.050
ICU2	923 (18.2)	384 (18.5)	539 (17.9)	
ICU6	787 (15.5)	314 (15.1)	473 (15.7)	
GCCU	479 (9.4)	190 (9.1)	289 (9.6)	
OIR	278 (5.5)	95 (4.6)	183 (6.1)	
VHDU	783 (15.4)	342 (16.4)	441 (14.7)	
LFU	652 (12.8)	248 (11.9)	404 (13.4)	
Day of the week				
Monday	745 (14.7)	313 (15.1)	432 (14.4)	0.513
Tuesday	752 (14.8)	310 (14.9)	442 (14.7)	
Wednesday	706 (13.9)	281 (13.5)	425 (14.1)	
Thursday	806 (15.9)	320 (15.4)	486 (16.2)	
Friday	704 (13.8)	274 (13.2)	381 (12.7)	
Saturday	642 (12.6)	261 (12.6)	381 (12.7)	
Sunday	730 (14.4)	321 (15.4)	409 (13.6)	
Category of call				
Medicine supply	4885 (96.1)	1985 (95.4)	2900 (96.5)	0.074
Medicine advice	200 (3.9)	95 (4.6)	105 (3.5)	

Medicine characteristics

**Figure 2:**
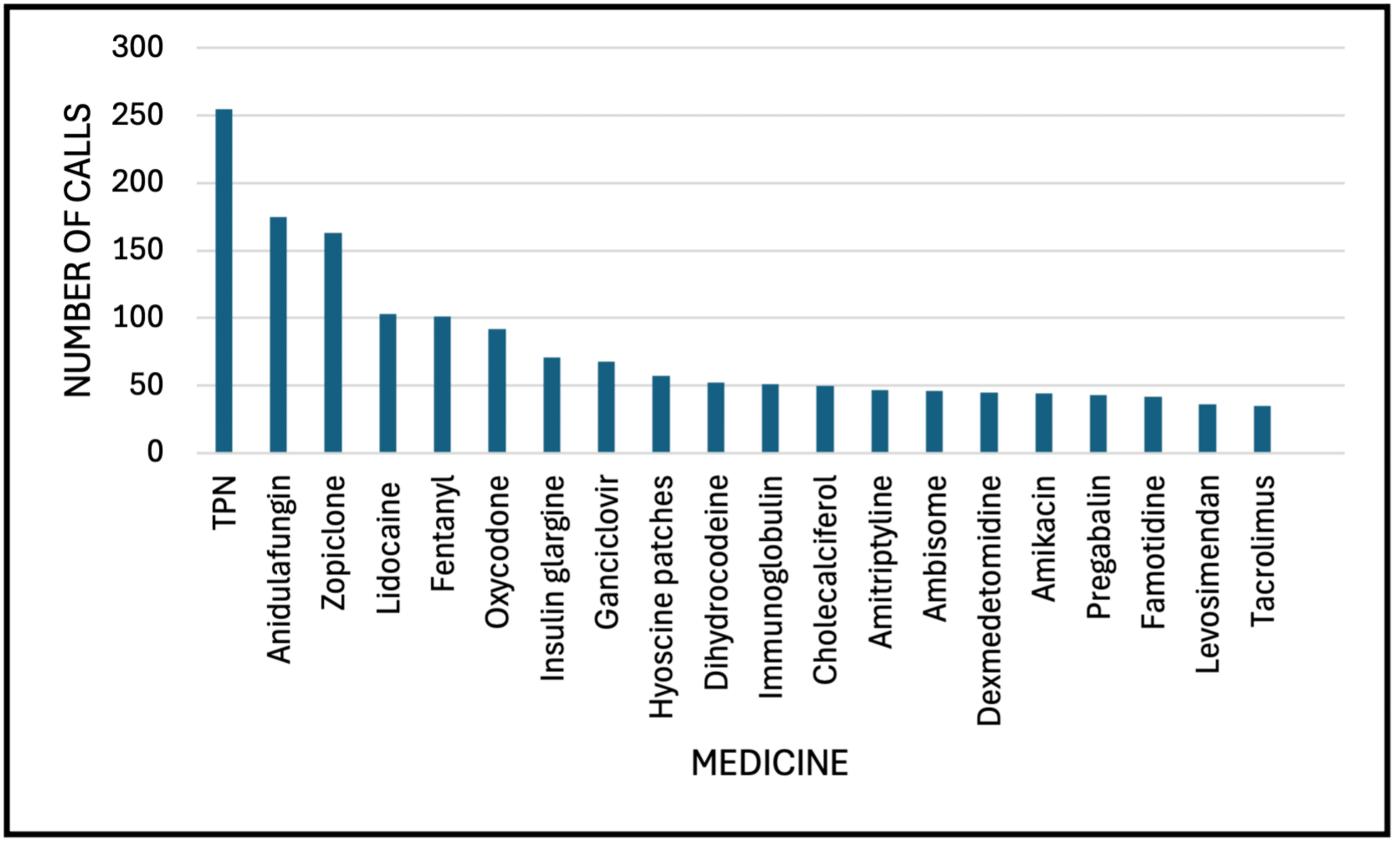
Top 20 most requested medicines out-of-hours from adult critical care TPN: total parenteral nutrition

There were 5915 medication requests for supply or advice during the study period. Total parenteral nutrition (TPN) was the most requested item (n=255; 4.3%), followed by anidulafungin (n=175; 3%), zopiclone (n=163; 2.8%), lidocaine (n=103; 1.7%), and fentanyl (n=101; 1.7%) (Figure 2). The most requested category of medicine was anti-infectives (antibacterials, antifungals, or antivirals) (n=921; 18.1%), followed by gastrointestinal (n=537; 10.6%) and cardiovascular (n=436; 8.6%) (Table 2). We compared the medicines' characteristics between evening and nighttime and found a higher proportion of calls during nighttime for all classes of drugs (p<0.001) and most routes of administration (p<0.001).

**Table 2:** Medicine characteristics of out-of-hours calls from adult critical care Other enteral routes include nasogastric (NG), nasojejunal (NJ), percutaneous endoscopic gastronomy (PEG), and radiologically inserted gastronomy (RIG). Topical routes include patches or creams. Anti-infectives include antibacterials and antivirals. Categorical data expressed as counts (% of counts) and compared using Pearson's χ^2^ test. *: p-values considered significant (p<0.05, 0.001)

Characteristic	All	5 pm to 9 pm	9 pm to 9 am	P-value
Class of drug				
Analgesia (non-opioid)	193 (3.8)	73 (3.5)	120 (4)	<0.001*
Analgesia (opioid)	348 (6.8)	109 (5.2)	239 (8)	
Anti-infective	921 (18.1)	374 (18)	547 (18.2)	
Cardiovascular	436 (8.6)	156 (17.5)	280 (9.3)	
Electrolyte	152 (3)	41 (2)	111 (3.7)	
Endocrine	336 (6.6)	147 (7.1)	189 (6.3)	
Gastrointestinal	537 (10.6)	310 (14.9)	227 (7.6)	
Immunosuppressant	89 (1.8)	40 (1.9)	49 (1.6)	
Neurological	135 (2.7)	46 (2.2)	89 (3)	
Psychiatric	189 (3.7)	64 (3.1)	125 (4.2)	
Respiratory	129 (2.5)	60 (2.9)	69 (2.3)	
Sedative	311 (6.1)	87 (4.2)	224 (7.5)	
Urological	45 (0.9)	18 (0.9)	27 (0.9)	
Vaccine	4 (0.1)	0 (0)	4 (0.1)	
Vitamins and minerals	98 (1.9)	37 (1.8)	61 (2)	
Other	939 (18.5)	404 (19.4)	535 (17.8)	
Route of administration				
Oral/other enteral routes	2169 (42.7)	910 (43.8)	1259 (41.9)	<0.001*
Intravenous	1832 (36)	699 (33.6)	1133 (37.7)	
Topical	318 (6.3)	162 (7.8)	156 (5.2)	
Subcutaneous	260 (5.1)	111 (5.3)	149 (5)	
Inhalation	156 (3.1)	77 (3.7)	79 (2.6)	
Intramuscular	15 (0.3)	5 (0.2)	10 (0.3)	
Other	334 (6.6)	116 (5.6)	218 (7.3)	

## Discussion

Statement of key findings

In this single-center retrospective observational study, we report characteristics of >5000 OOH calls received by resident pharmacists from adult critical care units in a large tertiary center, which is the largest study of its kind as far as we know. The main findings are the following: (1) most calls were for medication supply, with TPN the most requested item; (2) call volume was highest on Thursdays and during the winter period; (3) the most frequently requested class of drug was anti-infectives; (4) oral or other enteral route was the most required route of administration, followed by the IV route; and (5) the proportion of calls were highest during the nighttime period.

Interpretation

General Characteristics

We report a significant proportion of OOH calls to the resident pharmacist from healthcare professionals working in adult critical care units. Most calls were for medication supply, which concurs with other studies reporting OOH pharmacy activity in acute hospitals [2,10,15-17]. Call volume was highest from ICU1, which is unsurprising given it is a large general and emergency critical care unit with a high patient turnover [6]. January was the busiest month for OOH calls from adult critical care, as this is the month with the highest hospital activity in the calendar year [16,18]. The busiest day for OOH calls to the resident pharmacist in our study was Thursday, whereas in other studies, the highest volumes of OOH calls were received on Saturday and Sunday [15,16]. In our institution, an on-site adult critical care pharmacist service is provided from 9 am to 5 pm on Saturday and Sunday. It is therefore likely that a longer on-site service at weekends compared to other hospitals with a limited on-site pharmacist support lowers the on-call period and therefore lowers the number of OOH calls received [4,15,16]. Interestingly, most calls in our study took longer than 50 minutes to resolve, compared to other studies where most calls were resolved in less than 30 minutes [15,16]. It is possible that the time to resolve calls was longer in our study as it is common practice for resident pharmacists in our institution to deal with multiple issues at the same time including answering the phone, responding to queries from healthcare professionals at the hatch, and dispensing medicines on-site, including discharge medication and controlled drugs. However, in other institutions, the on-call pharmacy service is provided from home, and an emergency drug cupboard is likely available for nursing staff to access medicines [2,16]. There were no significant differences in the characteristics of calls when comparing evening and nighttime calls. This is surprising given the unequal time periods of evening (four hours) versus nighttime (12 hours) calls reported in our study. It is feasible that differences between the groups may be significant if the time periods (e.g., eight hours for evening and eight hours for nighttime) were equal.

The provision of a 24-hour resident pharmacist service is a positive step forward in providing seven-day service as recommended by NHS England and benefits patient outcomes through timely medicine dose optimization and a reduction in medication errors [19,20]. However, negative consequences of this include reports of anxiety, stress, and burnout in one study [21]. It is important that debriefing following a night shift is utilized to lower the incidence of untoward effects on the mental health of resident pharmacists [21]. Additionally, inappropriate calls have been reported to significantly contribute towards additional overpayments and frustration to pharmacists undergoing on-call activities; however, we did not report this in our study, which is a limitation [22,23]. Pharmacists participating in the on-call rota can be a financial and labor-intensive burden on departments as one pharmacist on a night shift cannot provide pharmaceutical care to patients for two days. Overall, the results suggest a nighttime (9 pm to 9 am) service is necessary given the volume of calls recorded; however, further work is required to optimize stock holdings of commonly used medicines on adult critical care units during daytime hours to reduce the burden on OOH services.

Medicine Characteristics

We report TPN as the most requested single item OOH, which corroborates with another study reporting characteristics of on-call activity in another UK hospital setting [17]. This is unsurprising, as TPN is an important high-risk medicine requiring considerations about IV line access and compatibility and is routinely stocked in the pharmacy department and procured from external providers. It is possible that a proportion of calls from healthcare professionals were due to delays in receiving TPN on critical care units, and this warrants further study. It is commonly used in adult critical care units to manage and treat malnutrition when there are contraindications to providing nutrition via the oral or enteral routes [24]. Malnutrition is very common in critically ill patients, occurring in 30-50% of hospitalized patients, and worsens over time in prolonged hospitalization [25,26]. Reasons for malnutrition requiring TPN in the ICU include patients undergoing surgical procedures (leading to gastrointestinal intolerance), infection, inflammation, and medications including antibiotics, opioids, sedatives, neuromuscular blocking drugs, and vasopressors [26-28].

Anti-infectives (including antibacterial, antifungal, and antiviral medicines) were the most frequently requested class of medicine OOH. There are several medicines in this class that are restricted to patient-specific supply owing to their high-risk nature requiring a clinical pharmacist review to ensure safety, efficacy, guideline/formulary compliance, and dose optimization in organ dysfunction. Some are high-cost drugs that require specific criteria to be met to ensure reimbursement from commissioners. Commonly requested high-risk anti-infectives OOH include anidulafungin, ceftazidime/avibactam, ceftolozane/tazobactam, ganciclovir, and liposomal amphotericin.

Most calls were for medicines to be administered via the oral/other enteral routes, followed by the IV route. Critical care patients often require a change in route of administration of medication from the oral route to via enteral feeding tube and parenterally [9]. Changing between routes of administration requires expert pharmacist knowledge to account for differences in bioavailability and release characteristics, necessitating changes to dose and frequency, and/or mitigate for enteral feed/drug interactions [9]. The administration of capsules or tablets through a variety of enteral routes can pose many challenges in adult critical care due to the prolonged-release or enteric-coated nature of the medicine [29]. Inappropriate administration can lead to occlusion of feeding tubes, a reduced medicine effect, or drug toxicity [29]. Administration of IV medication is high risk and associated with errors including issues with prescribing, administration, and documentation [30]. Examples of errors arising from IV medicine administration include wrong labelling, inappropriate diluent, and inappropriate rate and concentration [30]. It is therefore reassuring that a large proportion of calls received OOH are related to IV medications due to their high-risk nature.

Strengths and weaknesses

The strength of this study is that it is the largest study to date reporting the characteristics of OOH calls to the resident pharmacist from adult critical care units in the hospital setting, with access to a large database of OOH calls providing access to highly reliable granular data on medication name and route, context of the call, time period, and location. Our study has several weaknesses: First, this was a single-center retrospective analysis which is susceptible to confounders and limits generalizability. A potential confounding factor that may have contributed to the differences in characteristics in Tables 1-2 was the unequal time periods between groups (four hours for evening versus 12 hours for nighttime). It is feasible that the comparison in characteristics may be significantly different if time periods were equal between the groups. Additionally, we performed no adjustment for potential confounding factors which is a further limitation to the study design. Second, data was not reported on the category of healthcare professionals seeking medication supply or advice. Third, referrals of resident pharmacist OOH calls to the senior pharmacist on-call support service were not recorded. Fourth, we did not collect data on the proportion of calls relating to discharge medication or requests for controlled drugs. Fifth, the value or impact of advice given OOH was not measured. Sixth, unfortunately, we did not collect data on the proportion of calls that were deemed as inappropriate, defined as calls for non-urgent medication or advice where an available resource was available at ward level or repeated calls for the same medicines [22]. This was due to the volume of calls being analyzed and limited data on the clinical context of each call. This data would be useful as a quality improvement initiative to lower the frequency of unnecessary calls to the resident pharmacist. It is also feasible that a proportion of calls received by the resident pharmacist were not reported during the study period during times of increased workload. Additionally, in-person medicine queries from the pharmacy hatch may not be recorded using Smartpage®. These are further limitations to our study, and future work should explore this possibility through semi-structured interviews with resident pharmacists. It was also not clear from the Smartpage® data how the time to resolution of a call was calculated and whether this was documented by the resident pharmacist or calculated by some other means, limiting the reliability of this data point.

## Conclusions

The provision of OOH pharmacy services to adult critical care provides a timely supply and advice of critical medicines. However, no conclusions of impact on patient outcomes can be inferred due to the small observational nature of the study. Further work should explore the impact of calls on avoidable patient harm, the optimization of medicine supply to adult critical care units during daytime to reduce requests for non-urgent items, the economic analysis of the resident pharmacist on-call service, and whether the integration of artificial intelligence decision support could lower call volume.
